# The Effect of a Tectonic Stress Field on Coal and Gas Outbursts

**DOI:** 10.1155/2014/813063

**Published:** 2014-06-01

**Authors:** Fenghua An, Yuanping Cheng

**Affiliations:** ^1^National Engineering Research Center for Coal & Gas Control, Faculty of Safety Engineering, China University of Mining & Technology, Xuzhou 221116, China; ^2^State Key Laboratory of Coal Resources and Mine Safety, China University of Mining & Technology, Xuzhou 221116, China

## Abstract

Coal and gas outbursts have always been a serious threat to the safe and efficient mining of coal resources. Ground stress (especially the tectonic stress) has a notable effect on the occurrence and distribution of outbursts in the field practice. A numerical model considering the effect of coal gas was established to analyze the outburst danger from the perspective of stress conditions. To evaluate the outburst tendency, the potential energy of yielded coal mass accumulated during an outburst initiation was studied. The results showed that the gas pressure and the strength reduction from the adsorbed gas aggravated the coal mass failure and the ground stress altered by tectonics would affect the plastic zone distribution. To demonstrate the outburst tendency, the ratio of potential energy for the outburst initiation and the energy consumption was used. Increase of coal gas and tectonic stress could enhance the potential energy accumulation ratio, meaning larger outburst tendency. The component of potential energy for outburst initiation indicated that the proportion of elastic energy was increased due to tectonic stress. The elastic energy increase is deduced as the cause for a greater outburst danger in a tectonic area from the perspective of stress conditions.

## 1. Introduction

A coal and gas outburst is a dynamic failure that ejects coal mass containing high-pressure gas in a short time. As one main failure of underground coal resource exploitation, it causes severe damage in the major global producing nations [1, 2]. The knowledge available regarding the coal and gas outburst mechanism is still qualitative at present, and the ground stress, coal gas, and physical and mechanical properties of coal determine the formation and initiation of an outburst. Ground stress plays a primary role in coal mass that is broken before outburst initiation and provides potential energy for outburst initiation. Gas content of the coal seam is an essential element in an outburst [1] and is usually the main index for outburst danger. The coal gas aggravates the failure of coal mass before the outburst initiation. A portion of the coal gas would participate in outburst initiation. Afterwards, the enormous energy released by the coal gas would crush, strip, and carry the broken coal mass. The stress conditions can affect the coal mass failure, the potential energy accumulation for outburst initiation, and the outburst danger.

A key factor for underground resource exploitation is the in situ ground stress. The crustal stress on the stratum is mainly caused by gravity stress and tectonic stress. The gravity stress has a regular distribution. The maximum stress is the vertical stress and increases with depth linearly, and the lateral stress can be calculated with the coefficient of lateral pressure and is related to Poisson's ratio. The tectonic stress is caused by geotectonic movement and is mainly in the horizontal direction similar to the crustal movement. The results measured in situ show that the horizontal stress is greater than the vertical stress universally in shallow strata. The ratio between the horizontal stress and the vertical stress ranged from approximately 0.5 to 2 [3–10]. Generally speaking, in shallow depths, the tectonic forces have a significant effect on the horizontal stress and lead to a stress type of *σ*_*H*_≻*σ*_*v*_≻*σ*_*h*_ and to a stress type of *σ*_*H*_≻*σ*_*h*_≻*σ*_*v*_ in the intense tectonic zone. For greater depths, the impact of tectonics weakens, and the vertical stress increases and gradually changes the stress type to  *σ*_*v*_≻*σ*_*H*_≻*σ*_*h*_.

To control coal and gas outbursts [1], the effect of ground stress (especially the tectonic stress) on outbursts has been confirmed. Due to the susceptibility of open cracks due to stress, the permeability is significantly affected by the stress [11, 12]. The fracture connectivity of sheared coal was destroyed by tectonic events [13] and reduced the coal seam permeability [14]. As a result, the migration and storage of coal gas is strongly affected by the tectonic stress field and its evolution [14–16]. Additionally, the high tectonic stress has a primary role in the stress conditions of outburst areas, and the gas and ground stress abnormity in tectonic areas and neighboring areas is frequent [17]. Moreover, tectonism reduces the coal mass strength [18]. From these effects, tectonics has a critical influence on outburst occurrence and distribution [1, 18].

In addition, the ground stress measurement in outburst areas shows the characteristics of high ground stress. A study of the relationship between coal and gas outbursts and the ground stress of the Xie'er Coal Mine in the Chinese Huainan Coalfield showed a positive correlation [19]. The in situ stress distribution measured by Han et al. showed that with tectonic stress, the horizontal stress in outburst areas was greater in comparison with other areas [20]. In addition to the high ground stress, the spatial differentiation may be induced and may influence the deformation and failure of the coal and rock mass near the roadway. In the meanwhile, the study of roadway stability has shown that the magnitude and direction of the horizontal stress could affect the roadway stability significantly. The roadway that is parallel to the maximum horizontal stress has the best stability, and the roadway that is perpendicular to the maximum horizontal stress has the worst stability [21–23]. The cut-through roadway is most stable for the direction parallel or perpendicular to the maximum horizontal stress [23].

Previous research on the effect of the magnitude and distribution of ground stress on an outburst considers the predictability of an outburst occurrence in engineering. However, more research is required regarding the specific mechanism that causes an outburst. This paper focuses on the effect of the magnitude and distribution of ground stress and the influence of tectonics on an outburst. A numerical model considering the effect of coal gas is established. The coal mass failure and the potential energy accumulation surrounding the roadway is analyzed for different ground stress fields, and then the effect on the outburst tendency is analyzed.

## 2. Description of the Numerical Model

### 2.1. Governing Equation

As dual-porosity media, the stress state of the coal mass is affected by the gas pressure in pores and fractures. If compressive stress is positive, the effect of free gas on the coal stress state could be expressed by the effective stress equation of porous media [24]:

(1) $$\begin{array}{c} \sigma^{\prime }=\sigma -\left(\gamma_{m}p_{m}+\gamma_{f}p_{f}\right)\delta_{ij}, \end{array}$$

where *p*_*m*_ and *p*_*f*_ represent the gas pressure in the fracture and the coal matrix, respectively. The pore and fracture effective stress coefficients are represented by *γ*_*p*_ and *γ*_*f*_, respectively, and can be expressed as [24]

(2) $$\begin{array}{c} \gamma_{p}=\frac{K}{K_{m}}-\frac{K}{K_{s}}\\ \gamma_{f}=1-\frac{K}{K_{m}}, \end{array}$$

where *K* represents the bulk modulus of the fractured porous solid, that is, the coal mass; *K*_*m*_ represents the bulk modulus of the porous solid, that is, the coal matrix; and *K*_*s*_ represents the bulk modulus of the solid skeleton and is the coal skeleton without pores.

Coal mass expands after gas adsorption and can be compared to thermal expansion [25]. Considering the effect of coal mass, the relationship between the effective stress and strain can be

(3) $$\begin{array}{c} \sigma_{ij}^{\prime }=\lambda \varepsilon_{v}\delta_{ij}+2G\varepsilon_{ij}-K\varepsilon_{v}^{s}\delta_{ij}, \end{array}$$

where *λ* is the Lamé constant, *G* is the shear modulus, and *ε*_*Lv*_^*s*^ represents the maximum adsorption-induced volume strain. Research on the adsorption-induced deformation shows that the volumetric strain is approximately linear, is proportional to the adsorbed gas [26, 27], and can be approximated using the Langmuir-like equation [28]

(4) $$\begin{array}{c} \varepsilon_{v}^{s}=\frac{\varepsilon_{Lv}^{s}p_{m}}{p_{m}+p_{L}^{s}}, \end{array}$$

where *p*_*L*_^*s*^ represents the Langmuir pressure of the adsorption-induced strain.

Except for the induced deformation, the gas adsorption reduces the coal strength [29, 30]. The strength reduction is related to the adsorbed gas, and the reduction factor can be expressed with a linear relationship that assumes [28]

(5) $$\begin{array}{c} \alpha =\frac{\alpha_{0}p}{p+p_{L}}, \end{array}$$

where *α*_0_ represents the maximum reduction of the coal mechanical parameters.

The Mohr-Coulomb matching DP yield criterion is chosen for the failure criterion of the coal mass [31]:

(6) $$\begin{array}{c} F=\alpha_{\text{DP}}I_{1}+k_{\text{DP}}-\sqrt{J_{2}}, \end{array}$$

where *I*_1_ represents the first stress invariant, *J*_2_ represents the second deviator stress invariant, and *α*_DP_ and *k*_DP_ are identified by the cohesion  *C*  and the friction angle  *φ* with the inscribed circle way.

Coal mass is an elastic-plastic material with a strain-softening property. Hence, the strain-softening model is appropriate [32] and the mechanical parameters develop as the softening parameter [33]:

(7) $$\begin{array}{c} \omega =\left\{\begin{array}{cc} \omega_{0}-\left(\omega_{0}-\omega_{r}\right)\frac{\gamma^{p}}{\gamma^{p^{*}}}, & 0≺\gamma^{p}≺\gamma^{p^{*}}\\ \omega_{r}, & \gamma^{p}\geq \gamma^{p^{*}} \end{array}\right\}, \end{array}$$

where *ω* represents the mechanical parameter, *ω*_0_ represents the original parameters before the stress peak, *ω*_*r*_ represents the residual parameters at the residual zone, *γ* ^*p*^ represents the softening parameter, and *γ* ^p*^ represents the transition value of the softening parameter from which the residual behavior starts. A common softening parameter is the equivalent plastic shear strain [34]:

(8) $$\begin{array}{c} \gamma^{p}=\sqrt{\frac{2}{3}\left(\varepsilon_{1}^{p}\varepsilon_{1}^{p}+\varepsilon_{2}^{p}\varepsilon_{2}^{p}+\varepsilon_{3}^{p}\varepsilon_{3}^{p}\right)}, \end{array}$$

where *ε*_1_^*p*^, *ε*_2_^*p*^, and *ε*_3_^*p*^ are the principal plastic strains.

The dual-porosity model proposed by Warren and Root [35] is used primarily for gas migration in the coal seam. The adsorbed gas is desorbed from the pore wall and diffuse into fractures from the coal matrix with various diffusion forms. The dominant diffusion form is gaseous phase diffusion, the driving force of which is the gas density difference [36]. The coal matrix gas pressure evolves with time [28]:

(9) ∂pm∂t=(−1τ·(pm−pf)) ×((VLpmpL+pm−VLpL2pm(pL+pm)2+ϕmρp0)ρRTVM)−1,

where *τ* represents the adsorption time of the coal matrix; *V*_*L*_ represents the maximum adsorption volume of coal; *p*_*L*_  represents the Langmuir pressure of coal; *ϕ*_*m*_ represents the porosity of the coal matrix; *ρ* represents the bulk density of coal; *p*_0_ represents the atmospheric pressure; *R* represents the universal gas constant; *T* represents the gas temperature; and *V*_*M*_ represents the molar volume of gas.

As the source term, the gas in the coal matrix trades with the fracture, and the gas mass conservation equation for the fracture is

(10) ∂(ϕfpf)∂t−kμ∇·(pf·∇pf)−1τ ·(1−ϕf)(pm−pf)=0,

where *ϕ*_*f*_ represents the fracture porosity, *k* represents the coal seam permeability, and *μ* represents the gas viscosity coefficient (1.08*E* ^−5^ Pa·s for methane).

### 2.2. Approach and Parameters of the Model

**Figure 1:**
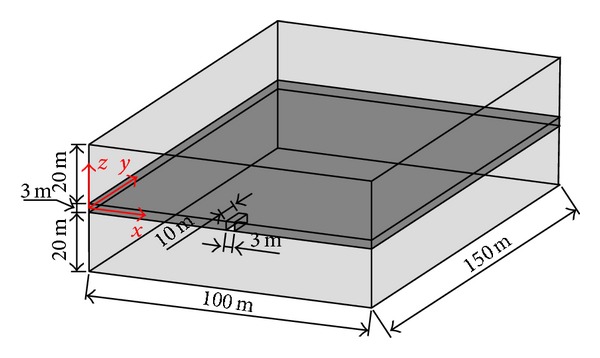
Geometric model.

The governing equations were performed by COMSOL Multiphysics using the solid and PED module. The model includes the coal seam in the middle and roof and floor rocks as the geometry model as shown in Figure 1. The finite element mesh consists of 74,692 tetrahedrons. The *xz* face (*y* = 0) at the beginning of the roadway excavation is the symmetry boundary. The *xy* face (*z* = 21.5) at the top of the model is the load boundary. The *xy* face (*z* = −21.5) at the bottom of the model is fixed. The two *yz* faces (*x* = 0; *x* = 100) and the *xz* face (*y* = 150) at the sides are the slip boundaries or the stress load boundaries according to the ground stress conditions. The excavation direction is in the *y* direction, and the roadway is 10 m long in the model.

Most outbursts occurred in the excavation advancement, and the coal mass in front of the working face is the most dangerous. The emphasis of this paper is on the analysis of the effect of ground stress conditions on coal mass deformation and failure, and the effect of roadway size on outburst danger was not analyzed. The mechanical properties of roof and floor rocks are simplified as hard elastoplastic material. The coal seam is a strain-softening elastoplastic material. The mechanical and gas migration parameters are obtained from previous studies and are shown in Table 1.

### 2.3. Conditions of Tectonic Stress and Coal Gas

In the tectonic area, the horizontal stress of the gravity stress field increases with the effect of tectonics and forms stress field types of *σ*_*v*_≻*σ*_*H*_≻*σ*_*h*_, *σ*_*H*_≻*σ*_*v*_≻*σ*_*h*_, and *σ*_*H*_≻*σ*_*h*_≻*σ*_*v*_. With an overlying strata load of 400 m deep and an average density of 2500 kg/m^3^(10 MPa) as the reference stress, the ground stress of the stress field types above is loaded in the model as shown in Table 2. The ground stress studied includes the gravity stress field (Case 1), the stress field influenced by tectonics (Cases 2 and 3), and the stress field strongly influenced by tectonics (Cases 4 and 5). The slip boundary was set as the gravity stress field in Case 1.

**Figure 2:**
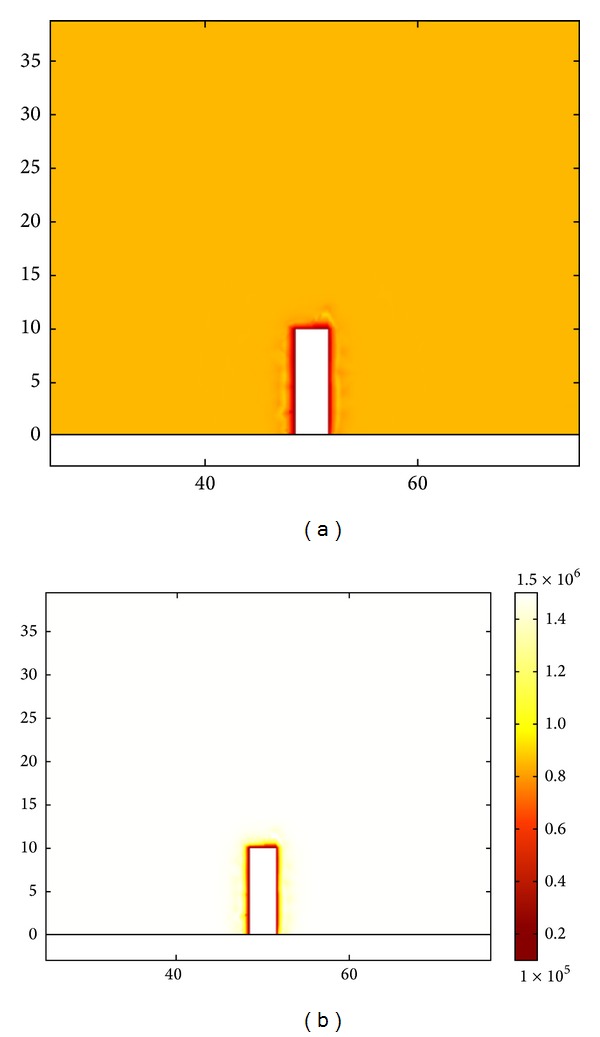
Distribution of coal gas pressure in fractures surrounding the roadway.

The difference between the coal seam roadway and the rock roadway is that the coal gas affects the stress state of the coal mass surrounding the roadway. With various stress and coal seam conditions, the gas pressure required for an outburst is different. 0.74 MPa (relative pressure) is recommended in China. The coal seams without coal gas and with gas pressure of 0.8 MPa and 1.5 MPa (9.3 m^3^/t and 12.7 m^3^/t correspondingly) are considered in this paper, and, with different stress conditions, the coal gas distributions surrounding the roadway are the same in the calculation as shown in Figure 2.  Figure 2(a) shows the fracture pressure distribution surrounding the roadway with an initial gas pressure of 0.8 MPa, and Figure 2(b) shows the fracture pressure distribution with an initial gas pressure of 1.5 MPa. The numbers beside the frame are coordinate values. The values in the legends represent gas pressure in fractures and the unit is Pa. The gas distribution is obtained after 0.5 days of gas migration to the coal wall with a permeability of 0.0025 mD.

## 3. The Outburst Tendency Analysis Based on Its Energy Requirement

The continuity condition required by the finite element method used above limits the analysis to broken and crush and is confined to the previous stage of dynamic failure of coal mass. Except for the strength failure as a precondition of the dynamic failure of the coal mass, the energy accumulated for an outburst initiation should be greater than the energy consumed. Based on this consideration, it can be deemed that the preparation of an outburst is an accumulation of potential energy for outburst initiation relative to the energy dissipated in coal mass crushing and stripping. The ratio of potential energy for outburst initiation and the energy consumed can be used to evaluate the outburst tendency. Gas pressure in the coal seam is much smaller than the stress, and the coal mass failure is mainly caused by the ground stress and the mining-induced stress. Gas pressure aggravates the failure and would crush and strip the broken coal mass in outbursts. The outbursts start in the failure zone of the coal mass. Therefore, the potential energy for outburst initiation in the failure zone is analyzed below and describes the outburst tendency using the ratio between the potential energy accumulated and the dissipation energy required in outburst initiation.

The energy requirement for coal and gas outbursts is that the potential energy for outburst initiation *E*_*i*_ should be greater than the energy consumed in the coal mass crush, stripping, and movement *E*_*w*_ [37]. The energy released in outbursts includes the elastic energy of the coal mass and the internal energy of the free gas in the fractures and the desorbed gas from the coal matrix. The energy that participates in outburst initiation includes the elastic energy stored in coal mass *E*_*e*_ and the internal energy of partial gas *E*_*g*_  due to the short time for outburst initiation. The gas involved occupies a small fraction of the coal gas. The energy consumed in movement and in broken coal in the follow-up process is provided by the sequentially desorbed gas from the coal matrix. Therefore, the potential energy for outburst initiation is defined as  *E*_*i*_ = *E*_*g*_ + *E*_*e*_. The energy consumed in outburst initiation is mainly for surface energy *E*_*b*_ and is consumed during the crushing and stripping of coal mass. *E*_*w*_ = *E*_*b*_ is assumed.

For the condition of three-dimensional stress with the generalized Hooke's law, the elastic energy per unit volume of coal mass is

(11) $$\begin{array}{c} e_{e}=\frac{1}{2E}\left[\sigma_{1}^{2}+\sigma_{2}^{2}+\sigma_{3}^{2}-2\upsilon \left(\sigma_{1}\sigma_{2}+\sigma_{2}\sigma_{3}+\sigma_{3}\sigma_{1}\right)\right]. \end{array}$$

The potential energy for outburst initiation by coal gas through expansion work can be calculated as

(12) $$\begin{array}{c} e_{g}=\frac{pV_{0}}{n-1}\left[{\left(\frac{p}{p_{0}}\right)}^{\left(n-1\right)/n}-1\right], \end{array}$$

where *V*_0_ represents the volume of coal gas involved and *n* represents the polytropic exponent. The gas expansion in outburst is a polytropic process, and *n* is approximately 1.25 [38]. The coal gas amount involved in outburst initiation is difficult to measure. In addition to free gas, research by Valliappan and Zhang showed that desorbed gas from the coal matrix played an important role [39]. As dual-porosity media, the gas in fractures is deemed to participate in the outburst initiation entirely, whereas the gas in the coal matrix participates partially. The potential energy for outburst initiation by coal gas is

(13) eg=pfVf0n−1[(pfp0)(n−1)/n−1]ρ +pmVm0n−1[(pmp0)(n−1)/n−1]ρ,

where *V*_*f*0_ represents the coal gas content in the fracture (mL/g) and *V*_*m*0_ represents the participant coal gas content from the coal matrix (mL/g).

The participant coal gas from the coal matrix varies [40] and is related to the gas content, coal particle size, and diffusion coefficient. The desorbed gas from the coal matrix in outburst initiation can be estimated by the desorption ratio  *λ*, and the participant coal gas from the coal matrix *V*_*m*0_ can be expressed as

(14) $$\begin{array}{c} V_{m0}=\lambda V_{m\infty }, \end{array}$$

where *V*_*m∞*_ represents the total desorbed gas of the coal matrix (mL/g). If the ambient pressure of the gas desorption is estimated according to atmospheric pressure, the total desorbed gas can be calculated based on the difference in the gas content in the coal matrix before outburst initiation and the gas content with the gas pressure as standard pressure. The participant internal energy would change from (17) to

(15) Eg=pfn−1pfϕfp0[(pfp0)(n−1)/n−1]+pmn−1×λ(pmϕmp0ρ+VLpmpL+pm−p0ϕmp0ρ−VLp0pL+p0)×[(pmp0)(n−1)/n−1]ρ.

The desorption ratio is estimated by the following method. The outburst initiation is a transient process, and the desorption amount of coal gas in a short time is linearly related to the square root of the desorption time and is expressed as [41]

(16) $$\begin{array}{c} \frac{V_{t}}{V_{\infty }}=\frac{6}{\sqrt{\pi }}\sqrt{\frac{Dt}{r_{p}^{2}}}, \end{array}$$

where *V*_*∞*_ represents the total desorbed gas (mL/g);  *D* represents the diffusion coefficient (cm^2^/s); and *r*_*p*_ represents the diffusion radius (cm). The adsorption time *τ* is related to the diffusion coefficient and the matrix size. The cubic model of the coal matrix can be estimated as [42]

(17) $$\begin{array}{c} \tau =\frac{L^{2}}{3\pi^{2}D}, \end{array}$$

where *L* represents the space between fractures (cm). If *r*_*p*_ is *L*/2, $\sqrt{D/r_{p}^{2}}$ can be obtained from (15). From the observation record of a coal and gas outburst test site at the Zhongliangshan Mine in China in 1977, the first thud in sonic record occurred without a change to other parameters at 1.5 s after the inducing blast, and then three thuds occurred at 2.5 s, 3.5 s, and 4 s [43]. The continuing time of outburst initiation is the average of the recorded occurrence time of the later three thuds. For the adsorption time in the model, the desorbed gas for outburst initiation is 0.677% of the total desorbed gas, and the desorption ratio is 0.677%.

The energy consumed in coal mass crushing is mainly the energy required by the increased surface and can be expressed by

(18) $$\begin{array}{c} e_{b}=s\cdot w\cdot \rho , \end{array}$$

where *s* represents the increased surface area (cm^2^/g) and *w* represents the specific energy (J/cm^2^). The study of the specific energy of coal showed that soft coal is proportional to the firmness coefficient, has an outburst danger with less specific energy than hard coal, and is approximately 2 × 10^−3^ J/cm^2^ [44, 45]. The additional specific surface area of the coal mass that is crushed by the hammer drop method was from 113 to 525 cm^2^/g [44], and the specific surface area of the fragment from the rock burst test was 200 cm^2^/g [46]. In this paper, the specific energy of 1 × 10^−3^ J/cm^2^ and the additional specific surface area of 150 cm^2^/g are used, and the surface energy consumed in outburst initiation is 0.15 J/g.

The outburst initiation requires the accumulation of more potential energy than is consumed. Moreover, the outburst was initiated in the failure zone. Hence, the potential energy accumulated in the failure zone of the coal mass is related to the necessary energy in coal mass crushing and is analyzed to indicate the outburst tendency by the ratio

(19) $$\begin{array}{c} R_{o}=\frac{\int_{V_{p}}e_{i}dV}{\int_{V_{p}}e_{b}dV}=\frac{\int_{V_{p}}\left(e_{e}+e_{g}\right)dV}{\int_{V_{p}}e_{b}dV}, \end{array}$$

where *V*_*p*_ is the plastic zone. When  *R*_*o*_ is greater than 1, the potential energy for outburst initiation is sufficient and indicates the danger of an outburst.

## 4. Results and Discussion

### 4.1. The Effect of Tectonic Stress on the Deformation and Failure of Coal Mass

The effect of horizontal stress on roadway stability is focused on the deformation and failure of coal walls and roofs [21–23]. Coal and gas outbursts occur in coal mass surrounding the roadway, and most outbursts occur in front of the heading face in excavation. The deformation and plastic zone surrounding the roadway with various ground stress conditions are analyzed, and the outburst tendency in the front is studied.

**Figure 3:**
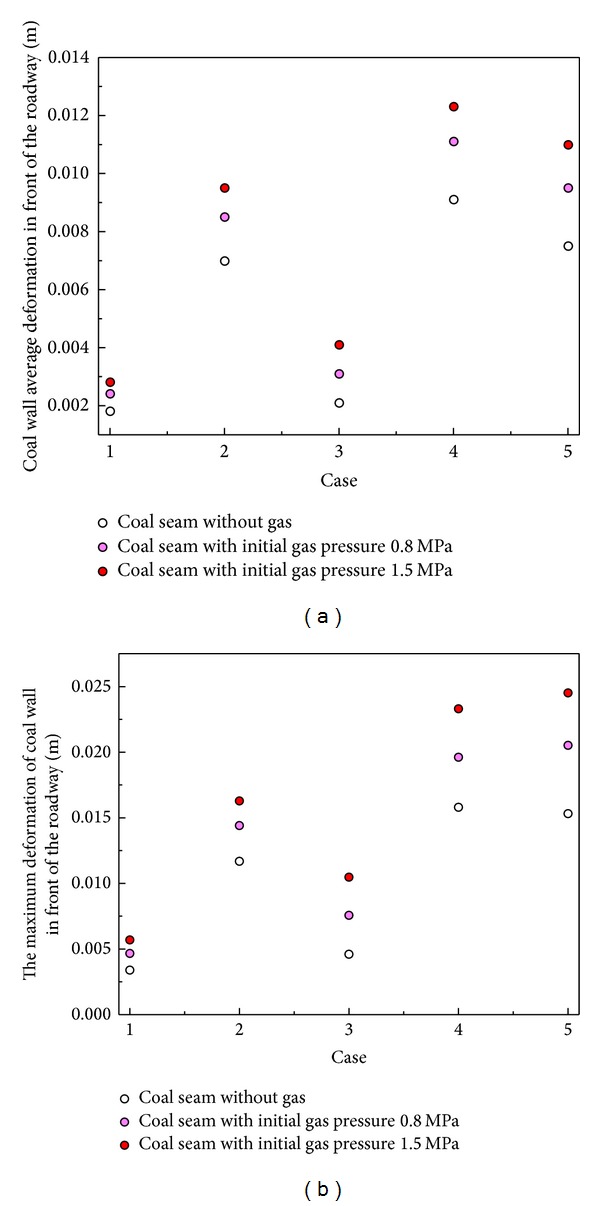
The deformation of the coal wall in front of the roadway.

The deformation of the coal wall is affected by the coal gas and stress conditions as shown in Figure 3. The tectonic stress increases the average and maximum deformation of the coal wall in front of the roadway, and the increase is affected by the distribution of tectonic stress. The coal mass deformation in front of the roadway is greater when the maximum horizontal stress is parallel to the direction of the roadway, but, as the tectonic stress increases, the effect of the stress distribution decreases. In addition, the coal gas has an important effect on the coal wall. For various stress conditions, the deformation increases as the coal gas increases.

**Figure 4:**
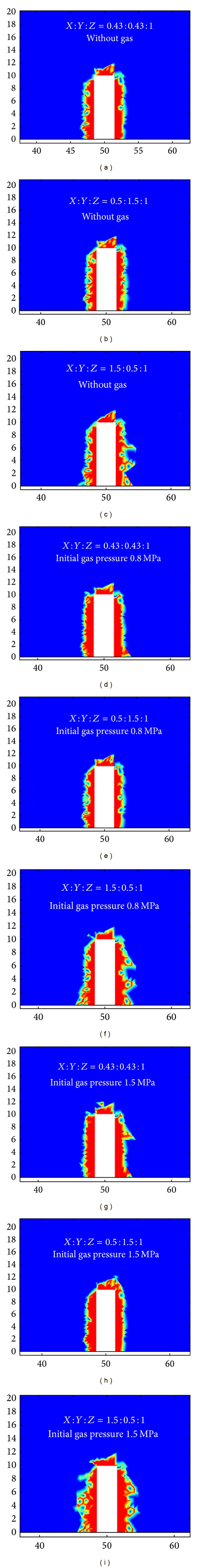
The plastic zone of the coal mass surrounding the roadway.

For various gas conditions, the size of the coal mass plastic zone in different stress conditions is shown in Table 3. The coal gas surrounding the roadway had an obvious effect on the plastic zone for pore pressure and strength reduction, causing a larger area with the same ground stress. The change in the ground stress condition affected the magnitude of the coal mass plastic zone and altered the distribution of the plastic zone. The plastic zone distribution for the middle cross-section of the coal seam with various ground stress fields is shown in Figure 4. The red zone is the plastic zone, and the blue zone is the elastic zone. With the alteration of coal mass failure, the outburst danger would be affected.

### 4.2. The Effect of Tectonic Stress on Outburst Tendency

**Figure 5:**
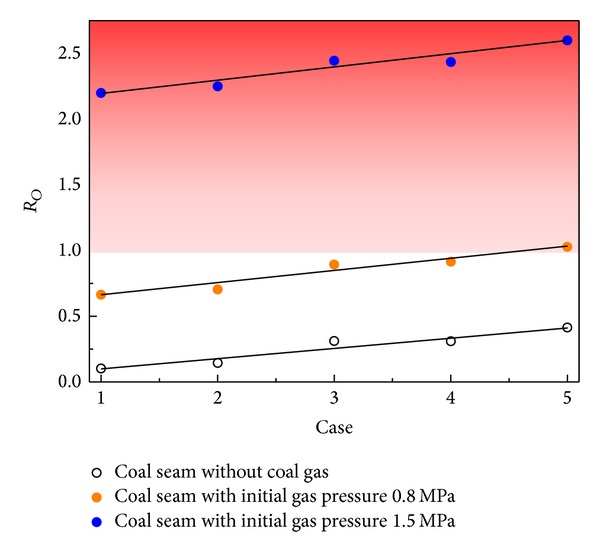
The effect of coal gas and tectonic stress on energy for outburst initiation in front of the roadway.

The energy accumulation for outburst initiation is affected by the coal gas and stress conditions as shown in Figure 5. The coal gas increased the potential energy for outburst initiation significantly, and, without coal gas, the accumulated energy had difficulty reaching the energy requirement for an outburst. The coal gas is the main influence factor for outburst initiation. Additionally, the tectonic stress has an important effect on the outburst tendency and is enhanced by tectonics as shown in Figure 5. For example, when the initial gas pressure is 0.8 MPa in the model, the tectonics could decide the occurrence of an outburst. This result verifies the previous ground stress measurement results that the horizontal stress was greater in the outburst area. Additionally, the stress distribution has an effect on the outburst. More potential energy is accumulated when the maximum horizontal stress is perpendicular to the roadway.

**Figure 6:**
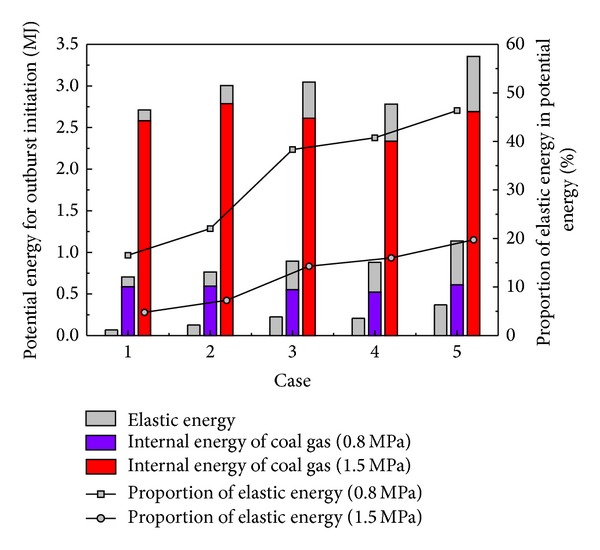
The elastic energy for outburst initiation in front of the roadway.

To further analyze the effect of tectonic stress, the energy composition for various tectonic stresses is studied as shown in Figure 6. The result showed that the proportion of elastic energy increases as the tectonic stress increase, and the proportion is greater when the maximum horizontal stress is perpendicular to the roadway. The elastic energy for outburst initiation that is increased by tectonic stress enhances the outburst tendency. Additionally, the distribution of the horizontal stress can alter the elastic energy. Additional elastic energy is held in excavation, when the maximum horizontal stress is perpendicular to the roadway and indicates a greater tendency for an outburst.

## 5. Conclusion

Affected by tectonics, the magnitude and spatial distribution of ground stress in mining areas are different. Previous research shows a close relationship between the outburst area and the tectonic stress. Tectonics and evolution cause the regional distribution of outbursts, and the outburst areas often have large tectonic stress. To analyze the effect of tectonic stress on outbursts, a numerical model of a roadway was established. In addition, a method for evaluating the outburst tendency was proposed based on the energy requirement for outburst initiation.

The deformation and failure of a coal mass in front of a roadway was analyzed. The results showed that the deformation and failure zone increased with coal gas. The pore pressure of free gas and the strength reduction of the adsorbed gas aggravated the coal mass failure and had a larger plastic zone with more gas in the coal seam. The tectonic stress enhanced the deformation and had an effect on the distribution of the plastic zone.

Based on the requirement of strength failure and energy for outburst initiation, the energy accumulation in the failure zone of coal mass is analyzed to show the outburst tendency using the ratio of potential energy for outburst initiation and the energy consumed. The results showed that coal gas was the dominant factor for energy accumulation in outburst initiation, and the increase in coal gas would enhance the outburst tendency significantly. Meanwhile, the ground stress change induced by tectonics has an important effect on an outburst. The outburst tendency increases as the tectonic stress increases and is verified in the previous measurement results in outburst areas. Moreover, the change in the composition of potential energy for outburst initiation showed that this result was due to the increase in elastic energy.

## Figures and Tables

**Table 1:** Model parameters.

Parameters	Value
Elastic modulus of coal (*E*_*c*_, GPa)	2 [47]
Poisson ratio of coal (*υ*_*c*_)	0.3 [47]
Bulk modulus of the coal matrix (*K*_*m*_, GPa)	7.5 [48]
Bulk modulus of the coal skeleton (*K*_*s*_, GPa)	8.9 [28]
Maximum adsorption-induced volume strain for CH_4_ (*ε*_*Lv*_^*s*^)	0.012
Langmuir pressure of adsorption-induced volume strain for CH_4_ (*p*_*L*_^*s*^, MPa)	1
Initial cohesion of coal without gas (*C*_*c*_, MPa)	0.923
Residual cohesion of coal without gas (*C*_c_*, MPa)	0.692
Friction angle of coal (*φ*_*c*_, °)	30 [47]
Transition value of coal softening parameter (*γ* ^*p*∗^, %)	2
Bulk density of coal (*ρ*_*c*_, kg/m^3^)	1300
Bulk density of rock (*ρ*_*r*_, kg/m^3^)	2500
Elastic modulus of rock (*E*_*r*_, GPa)	20
Cohesion of rock (*C*_*r*_, MPa)	20
Friction angle of rock (*φ*_*r*_, °)	40
Poisson ratio of rock (*υ*_*r*_)	0.3
Temperature of coal seam (*T*, K)	293
Initial permeability of coal seam (*k*_0_, mD)	0.0025 [38]
Initial fracture porosity (*ϕ*_*f*0_, %)	0.1 [49]
Matrix porosity (*ϕ*_*m*_, %)	6 [38]
CH_4_ Langmuir volume (*V*_*L*_, m^3^/t)	20
CH_4_ Langmuir pressure (*P*_*L*_, MPa)	1
Adsorption time (*τ*, day)	11.7 [47]

**Table 2:** Conditions of ground stress and coal gas in the model.

Case	Ground stress in each direction*			Initial gas pressure in coal seam/MPa		
	*X* direction	*Y* direction	*Z* direction	I	II	III
1	Slip boundary		1	0	0.8	1.5
2	0.5	1.5	1			
3	1.5	0.5	1			
4	1.5	2	1			
5	2	1.5	1			

*The values of ground stress in the table are shown with 10 MPa as a reference.

**Table 3:** The plastic zone of the coal mass in front of the roadway (m^3^).

	Case 1	Case 2	Case 3	Case 4	Case 5
Coal seam without gas	3.40	4.44	3.70	3.41	4.57
Coal seam with initial gas pressure 0.8 MPa	5.44	5.55	5.13	4.93	5.67
Coal seam with initial gas pressure 1.5 MPa	6.32	6.85	6.39	5.86	6.62
